# Medical Properties of Vegetables

**Published:** 1891-09

**Authors:** 


### Medical Properties of Vegetables.

Spinach has a direct effect upon the kidneys.

The common dandelion, used as greens, is excellent for the same trouble.

Asparagus purges the blood. Celery acts admirably upon the nervous system, and is a cure for rheumatism and neuralgia.

Tomatoes act upon the liver.

Beets and turnips are excellent appetizers.

Lettuce and cucumbers are cooling in their effects upon the system.

Onions, garlic, leeks, olives and shallots, all of which are similar, possess medical virtues of a marked character, stimulating the circulatory system and the consequent increase of the salivary and gastric juices, promoting digestion.

Red onions are an excellent diuretic, and the white ones are recommended to be eaten raw as a remedy for insomnia. They are tonic and nutritious. A soup made from onions is regarded by the French as an excellent restorative in debility of the digestive organs.—*Scientific American.*

"MEDICINE consists in addition and subtraction, the addition of those things which are deficient, and the subtraction of those things which are redundant; and he who practices this is the best physician, and he whose practice is furthest from it, is the furthest removed from a knowledge of the art."—HIPPOCRATES.

"HAPPY is he, therefore, who judges correctly and knows men—himself included—intuitively, or from early training, whose feelings are balanced by his intellect, and who is thoroughly in earnest in striving to do that which is good. But he whose environment has not been, or is not so favorable, and still who chooses to do that only which is right, is the truest exponent of free agency, whose living has made the world better than he found it, and who impresses most deeply his identity on the brightest pages of the history of the country, or the community in which he lives."—Extract from PROF. J. S. CASSIDY's address to graduating class at Cincinnati, March 11, 1891.

